# Supplementary material for: From sequence to enzyme mechanism using multi-label machine learning
Source: BMC Bioinformatics. 2014 May 19;15:150. doi: 10.1186/1471-2105-15-150 (PMC4229970; doi:10.1186/1471-2105-15-150)
Supplement: Additional file 2 — Java code of ml2db. Additional file ml2db_code.tar.gz contains the Java source code to run the multi-label machine learning experiments and save the results to database. The code’s Javadoc is included. [file 1471-2105-15-150-S2.zip › additional file 2/ml2db/utils/doc/cern/jet/random/AbstractDistribution.html]

AbstractDistribution


JavaScript is disabled on your browser.


- Overview
- Package
- Class
- Use
- Tree
- Deprecated
- Index
- Help

- Prev Class
- Next Class

- Frames
- No Frames

- All Classes

- Summary:
- Nested |
- Field |
- Constr |
- Method

- Detail:
- Field |
- Constr |
- Method


cern.jet.random

## Class AbstractDistribution

- java.lang.Object
- - cern.colt.PersistentObject
  - - cern.jet.random.AbstractDistribution

- All Implemented Interfaces:
  :   DoubleFunction, IntFunction, java.io.Serializable, java.lang.Cloneable

  Direct Known Subclasses:
  :   AbstractContinousDistribution

  ---

    

  ```
  public abstract class AbstractDistribution
  extends PersistentObject
  implements DoubleFunction, IntFunction
  ```

  Abstract base class for all random distributions.
  A subclass of this class need to override method nextDouble() and,
  in rare cases, also nextInt().

  Currently all subclasses use a uniform pseudo-random number generation engine
  and transform its results to the target distribution. Thus, they expect such
  a uniform engine upon instance construction.

  `MersenneTwister` is recommended as uniform
  pseudo-random number generation engine, since it is very strong and at the
  same time quick. `makeDefaultGenerator()` will conveniently construct
  and return such a magic thing. You can also, for example, use
  `cern.jet.random.engine.DRand`, a quicker (but much weaker) uniform
  random number generation engine. Of course, you can also use other strong
  uniform random number generation engines. Paul Houle's RngPack package
  contains some such beasts; `Ranecu`,
  `Ranecu` and
  `Ranlux` are strong well analyzed
  research grade uniform pseudo-random number generators with known periods.
  More info on RngPack can be found  here.

  **Ressources on the Web:**

  Check the Web version of the CERN Data Analysis Briefbook . This will clarify the definitions of most distributions. Also consult the StatSoft Electronic Textbook - the definite web book. **Other useful ressources:** Another site and yet another site describing the definitions of several distributions. You may want to check out a Glossary of Statistical Terms. The GNU Scientific Library contains an extensive (but hardly readable) list of definition of distributions. Use this Web interface to plot all sort of distributions. Even more ressources: Internet glossary of Statistical Terms, a text book, another text book. Finally, a good link list Statistics on the Web.

  Version:
  :   1.0, 09/24/99

  Author:
  :   wolfgang.hoschek@cern.ch

  See Also:
  :   `cern.jet.random.engine`,
      `edu.cornell.lassp.houle.RngPack`,
      `cern.jet.random.engine.Benchmark`,
      `cern.jet.random.Benchmark`,
      Serialized Form

- - ### Method Summary

    Methods

    | Modifier and Type | Method and Description |
    | --- | --- |
    | `double` | `apply(double dummy)` Equivalent to nextDouble(). |
    | `int` | `apply(int dummy)` Equivalent to nextInt(). |
    | `java.lang.Object` | `clone()` Returns a deep copy of the receiver; the copy will produce identical sequences. |
    | `static RandomElement` | `makeDefaultGenerator()` Constructs and returns a new uniform random number generation engine seeded with the current time. |
    | `abstract double` | `nextDouble()` Returns a random number from the distribution. |
    | `int` | `nextInt()` Returns a random number from the distribution; returns (int) Math.round(nextDouble()). |

    - ### Methods inherited from class java.lang.Object

      `equals, getClass, hashCode, notify, notifyAll, toString, wait, wait, wait`

- - ### Method Detail


    - #### makeDefaultGenerator

      ```
      public static RandomElement makeDefaultGenerator()
      ```

      Constructs and returns a new uniform random number generation engine
      seeded with the current time. Currently this is
      `MersenneTwister`.


    - #### apply

      ```
      public double apply(double dummy)
      ```

      Equivalent to nextDouble(). This has the effect that
      distributions can now be used as function objects, returning a random
      number upon function evaluation.

      **Specified by:**
      :   `apply` in interface `DoubleFunction`

      Parameters:
      :   `dummy` - argument passed to the function.

      Returns:
      :   the result of the function.


    - #### apply

      ```
      public int apply(int dummy)
      ```

      Equivalent to nextInt(). This has the effect that distributions
      can now be used as function objects, returning a random number upon
      function evaluation.

      **Specified by:**
      :   `apply` in interface `IntFunction`

      Parameters:
      :   `dummy` - argument passed to the function.

      Returns:
      :   the result of the function.


    - #### clone

      ```
      public java.lang.Object clone()
      ```

      Returns a deep copy of the receiver; the copy will produce identical
      sequences. After this call has returned, the copy and the receiver have
      equal but separate state.

      **Overrides:**
      :   `clone` in class `PersistentObject`

      Returns:
      :   a copy of the receiver.


    - #### nextDouble

      ```
      public abstract double nextDouble()
      ```

      Returns a random number from the distribution.


    - #### nextInt

      ```
      public int nextInt()
      ```

      Returns a random number from the distribution; returns
      (int) Math.round(nextDouble()). Override this method if
      necessary.


- Overview
- Package
- Class
- Use
- Tree
- Deprecated
- Index
- Help

- Prev Class
- Next Class

- Frames
- No Frames

- All Classes

- Summary:
- Nested |
- Field |
- Constr |
- Method

- Detail:
- Field |
- Constr |
- Method
